# Correction: Sun et al. Puerarin-V Improve Mitochondrial Respiration and Cardiac Function in a Rat Model of Diabetic Cardiomyopathy via Inhibiting Pyroptosis Pathway Through P2X7 Receptors. *Int. J. Mol. Sci.* 2022, *23*, 13015

**DOI:** 10.3390/ijms27146161

**Published:** 2026-07-10

**Authors:** Shuchan Sun, Awaguli Dawuti, Difei Gong, Ranran Wang, Tianyi Yuan, Shoubao Wang, Cheng Xing, Yang Lu, Guanhua Du, Lianhua Fang

**Affiliations:** 1 State Key Laboratory of Bioactive Substances and Functions of Natural Medicines, Institute of Materia Medica, Chinese Academy of Medical Sciences and Peking Union Medical College, Beijing 100050, China; sunsc@imm.ac.cn (S.S.);; 2 Beijing Key Laboratory of Drug Targets Identification and Drug Screening, Institute of Materia Medica, Chinese Academy of Medical Sciences and Peking Union Medical College, Beijing 100050, China; 3 Beijing Key Laboratory of Polymorphic Drugs, Institute of Materia Medica, Chinese Academy of Medical Sciences and Peking Union Medical College, Beijing 100050, China

**Error in Corresponding Author Name**

In the original publication [1], there was a mistake in the corresponding author’s name as published. The spelling of the published corresponding author’s name was wrongly written as “Lianghua Fang”. The correct spelling is “Lianhua Fang”.

**Error in Figure 4**

In the original publication [1], there was a mistake in Figure 4A (The situation of P–V loop of different groups) as published. The P–V loop image currently labeled as “Puer-V-200 mg/kg” in the published article was mistakenly replaced as the image of “control” during file preparation. It should be noted that this error only affects the representative schematic image, and all quantitative data and statistical analyses associated with Figure 4 remain accurate and unchanged. The corrected Figure 4 appears below. 

**Figure 4:**
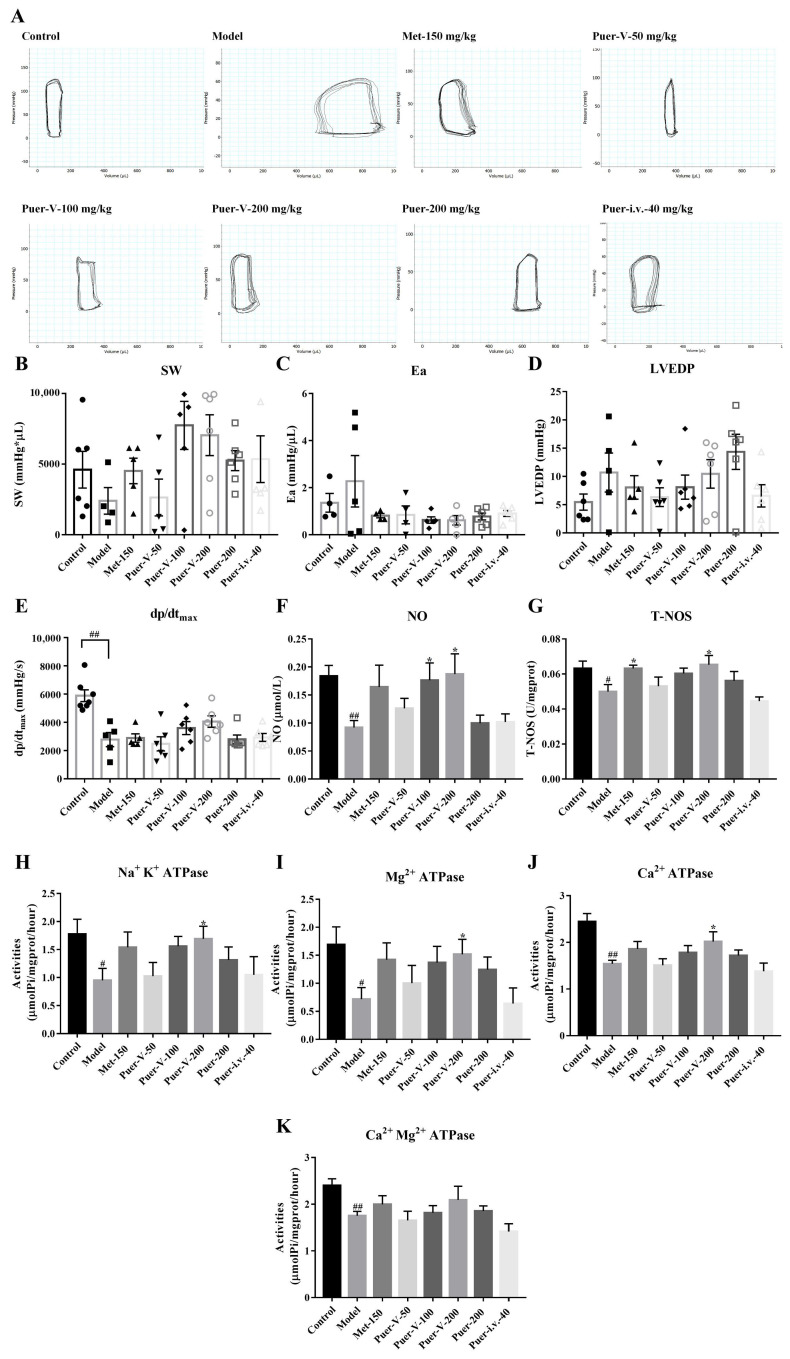
Puerarin-V enhanced the function of hemodynamics and left ventricular. (**A**) The situation of P–V loop of different groups, (**B**) LVSW, (**C**) Ea, (**D**) LVEDP, and (**E**) dp/dt_max_. (**F**) The content of NO. (**G**) The activity of NOS. (**H**) The activity of Na^+^-K^+^-ATPase. (**I**) The activity of Mg^2+^-ATPase. (**J**) The activity of Ca^2+^-ATPase. (**K**) The activity of Ca^2+^Mg^2+^-ATPase in diabetic cardiomyopathy rats. The data are represented by mean ± SEM (n = 6–8). ^#^ *p* < 0.05 and ^##^ *p* < 0.01 vs. control group. * *p* < 0.05 vs. model group.

**Error in Figure 5**

In the original publication [1], there was a mistake in Figure 5A (Representative echocardiographic M-mode records of left ventricular wall and Doppler images of aortic flow) as published. In the PW mode of echocardiogram results, the representative picture of the metformin group was presented incorrectly. It should be noted that this error only affects the representative schematic image, and all quantitative echocardiographic parameters and statistical results in Figure 5 remain accurate and unchanged. The corrected Figure 5 appears below. 

**Figure 5:**
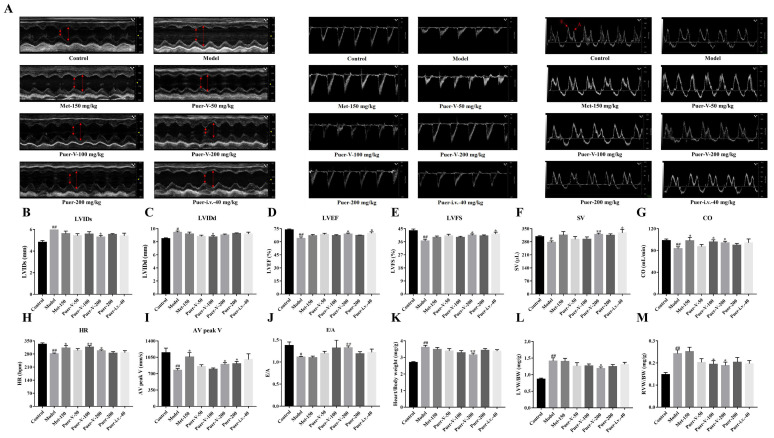
Puerarin-V ameliorated cardiac function in the DCM rats. (**A**) Representative echocardiographic M-mode records of left ventricular wall and Doppler images of aortic flow. Effects of Puerarin-V on LVIDs (**B**), LVIDd (**C**), LVEF (**D**), LVFS (**E**), SV (**F**), CO (**G**), HR (**H**), AV peak V (**I**), and E/A ratio (**J**). (**K**) The heart weight/body weight ratio. (**L**) The left ventricular weight/body weight ratio. (**M**) The right ventricular weight/body weight ratio. The data are represented by mean ± SEM (n = 6–8). ^#^ *p* < 0.05 and ^##^ *p* < 0.01 vs. control group. * *p* < 0.05 and ** *p* < 0.01 vs. model group.

The authors state that the scientific conclusions are unaffected. This correction was approved by the Academic Editor. The original publication has also been updated.
